# Seminal Extracellular Vesicles as Molecular Modulators of Male Reproductive Function

**DOI:** 10.1111/rda.70217

**Published:** 2026-09-30

**Authors:** Heriberto Rodriguez‐Martinez, Jordi Roca, Fernando Peña Vega, Emilio A. Martinez

**Affiliations:** ^1^ Department of Biomedical & Clinical Sciences (BKV), BKH/Obstetrics & Gynaecology, Faculty of Medicine and Health Sciences Linköping University Linköping Sweden; ^2^ Department of Medicine and Animal Surgery, Faculty of Veterinary Medicine, International Excellence Campus for Higher Education and Research “Campus Mare Nostrum” University of Murcia Murcia Spain; ^3^ Laboratory of Equine Reproduction and Equine Spermatology Veterinary Teaching Hospital Caceres Spain

**Keywords:** ARTs, domestic animals, ejaculate, extracellular vesicles, female genital tract, fertility, human, male genital tract, nanoplastics, seminal plasma

## Abstract

Extracellular vesicles (sEVs) contained in seminal plasma (SP) maintain, in concert with free SP‐components (proteins, hormones, cytokines, metabolites and antioxidants) under an endocrine umbrella the functional in vivo homeostasis of ejaculated spermatozoa. The sEVs define a generic mixture of nanovesicles delivered by male epithelial cells, from the testis to the accessory glands, where their lipid membrane encloses and protects a load of proteins, nucleic acids, enzymes, and lipids. This cargo can, following molecular uploading to target cells, dynamically interplay with spermatozoa allowing membrane remodelling pre‐fertilization. It can also interact with the lining epithelia of the female internal reproductive tract to induce gene expression changes, modifying ovulation timing and the female immune system leading to tolerate paternal epitopes. This review critically summarizes experimental evidence of the function of the sEVs on fertility, for their primary relevance as *diagnostic and fertility biomarkers* envisioning possible use of molecular components to select breeding sires, as well as for eventual *additives* to improve sperm cryosurvival or refine reproductive strategies, including in vitro ARTs. Since methodological stringency is a priority, particular attention is given to sEVs isolation, characterization and experimentation, bound with eventual risks of nanoplastic contamination, keeping view of the delicate balance between SP‐ and sEVs‐molecules and their concerted effects on male fertility.

## 1 Introduction

The utmost goal of reproduction, whether we consider theriogenology or human reproduction, is to reach possible optimal fertility (Zuidema et al. 2021). Fertility is burdened, and for some species and innumerable reasons, highly compromised (Huang et al. 2023; Khan et al. 2024; Ribas‐Maynou et al. 2023), calling for early diagnosis and the design and application of diverse treatment‐like tools (Ribas‐Maynou et al. 2023). Andrologists have thus examined semen, the functional male end‐product (Rodriguez‐Martinez 2014), looking for accurate diagnostic biomarkers for dysfunction and (in)fertility (Llavanera 2024; Rana et al. 2024).

The priest and researcher Lazzaro Spallanzani was in the late 1700s the one providing the first concrete evidence for the sperm role in amphibian fertilization, and also being the first one believing (as the “ovist” he was) that the apparently sperm‐free fluid (which he named “vapour”) would trigger embryo growth (Andrade‐Rocha 2017). Quite some time passed, becoming evident that his concept had a bearing; that semen would merely act via its sperm genomic vectors to deliver paternal DNA at fertilization is a persistent misconception. Semen contains, in the acellular composite fluid named seminal plasma (SP) an integral actor in reproduction via free and particulate components, highlighted by the growing amount of literature published on the role of the SP (Fernandez‐Fuertes 2023; Macay et al. 2026; Rodriguez‐Martinez et al. 2021) and the dissected roles of seminal extracellular vesicles (sEVs); a generic term embracing a plethora of particulate SP‐components (Jeppesen et al. 2024; Martínez‐Díaz, Parra, Montesdeoca, et al. 2024).

The dynamic interactions between the SP‐sEVs, spermatozoa and the lining epithelia of either male‐ or female reproductive tracts has been experimentally explored and reviewed over the past years (Alcântara‐Neto et al. 2020; Fazeli et al. 2025; Llavanera 2024; Parra et al. 2023; Ragni 2025; Roca et al. 2022; Rodriguez‐Martinez et al. 2021; Rodriguez‐Martinez and Roca 2022). This SP‐EVs axis displays a highly effective molecular biosystem, dependent on hormone signalling and redox homeostasis (Dlamini et al. 2025; Sengupta et al. 2026), reflecting male reproductive health (Wang et al. 2025; Xie et al. 2025). The SP contains a multitude of bioactive molecules including hormones, proteins (including enzymes), metabolites, antioxidants, and nucleic acids (DNA and diverse RNAs), either free or contained within membrane‐protected nanometre‐size sEVs loaded with lipids, proteins (including enzymes), and nucleic acids (DNA and diverse RNAs), as amply reviewed (Jeppesen et al. 2024; Neyroud et al. 2021; Rodriguez‐Martinez and Roca 2022; Voros et al. 2025; Wang et al. 2025; Xie et al. 2025). All these SP‐sEVs molecules would—by delivery—operate through dynamic crosstalk to shield spermatozoa from oxidative insults, preserving their functional homeostasis. In addition, they act as regulatory signals for key reproductive processes, as pre‐ and post‐fertilization events, ovulation timing, and modulation of the female immune response towards maternal tolerance to paternal epitopes, throughout pregnancy (Rodriguez‐Martinez et al. 2024; Xie et al. 2025; Xu, Xie, et al. 2024; Xue et al. 2024). This evidence suggests some SP‐sEVs molecules could be suitable biomarkers of sperm function (Xie et al. 2025; Xu, Zhang, et al. 2024) and even of male fertility (Dlamini et al. 2025; Voros et al. 2025). Current research is, however, hampered by a shortage of stringency regarding the isolation and characterization of sEVs. Considerable efforts are, therefore, directed to improve current methodologies and develop new approaches to advance in the identification of suitable diagnostic biomarkers of sperm (dys)function, resilience to sperm translational handling, including cryopreservation, and—ultimately—if biomarkers can be mapped to select best sires: fertility prognosis. In addition, these advances may support ARTs outputs, using native or enriched EVs as additives (Dlamini et al. 2025; Zhou et al. 2025).

The present review critically summarizes experimental data on the role of SP‐sEVs on fertility, with a certain overweight on some species, owing to the research profile of the authors. It focuses on *diagnostic and fertility biomarkers*, envisioning possible use of molecular components in sEVs to select breeding sires, as well as for eventual *additives* to improve cryosurvival or refine reproductive strategies including in vitro ARTs, keeping view of the delicate balance between SP‐sEVs components, to concert effects. Methodological stringency is a priority, with attention given to isolation, characterization and experimentation with sEVs. Likewise, a call for caution is made regarding the presence of nanoplastics.

## 2 Seminal Plasma: An Endocrine‐Paracrine Hub

Comparative clinical and experimental evidence marks the relevance of SP in maintaining sperm homeostasis during sperm transport through the female reproductive tract, regulating sperm‐female interactions, and balancing inflammatory responses against pathogens while promoting tolerance to paternal antigens, even beyond fertilization (Alvarez‐Rodriguez et al. 2024; Rodriguez‐Martinez et al. 2024). The SP provides both an electrolyte‐defined vehicle for spermatozoa, a source of substrates, as well as a particular proteome that carries multiple signalling molecules, including hormones. These components are able to interact with secretions and epithelial cells of the internal female genital tract, including antigen‐presenting cells (APCs), that initiate gene‐responses at the immune system level (Alvarez‐Rodriguez, Atikuzzaman, et al. 2019; Barranco et al. 2020; Bustamante‐Filho et al. 2022; Dlamini et al. 2025; Gardela et al. 2022; Harrison et al. 2024; Nongbua et al. 2020; Martinez et al. 2021; Xie et al. 2025). The SP is rich in inorganic ions (Ca^2+^, Zn^2+^, Mg^2+^, Na^+^, K^+^) essential to sperm homeostasis and reactivity (Martinez et al. 2021; Sengupta et al. 2026). In addition, SP contains testosterone, oestradiol, oxytocin, cortisol, inhibin, leptin, insulin and adiponectin, all measurable in the ejaculate of various species and biologically active within the female reproductive tract. These hormones also influence sperm homeostasis and reactivity, including sperm cell channels (Sengupta et al. 2026; Zhang, Liang, et al. 2024) and the molecular cargo of sEVs which, in pigs carry testosterone (P. Parra, personal communication) making some events testosterone‐dependent (Ma et al. 2018). Seminal hormones mirror the systemic metabolic milieu in both sexes, acting in concert with paracrine mediators (growth factors, cytokines, prostaglandins) to shape local microenvironments and immune responses during sperm maturation, transport, colonization of reservoirs and fertilization, ultimately influencing fertility and even male health (Padilla et al. 2021; Sengupta et al. 2026; Xie et al. 2025). For instance, the environment affects the content of miRNAs in sEVs, as seen in heat‐stressed bulls (Alves et al. 2021). Moreover, the SP protects spermatozoa from handling stress during extension or cryopreservation (Rodriguez‐Martinez et al. 2024).

What role is then played by the various SP molecular components? Do they act individually? Maybe, but they seem to act in concert, both towards the spermatozoa and the inseminated female (Roca et al. 2022; Rodriguez‐Martinez and Roca 2022). The SP‐proteome and peptidome is probably the most studied, particularly in cattle and pigs (Bustamante‐Filho et al. 2022; Xie et al. 2025). SP‐proteins had been characterized for their temporal distribution and ‐through transcriptomic analyses‐ their presence and relative concentrations related to sperm homeostasis during sperm transport and to fertilization, embryo development and fertility (Martinez et al. 2019; O'Leary et al. 2011; Padilla et al. 2020; Pérez‐Patiño et al. 2018; Roca et al. 2020; Rodríguez‐Martínez et al. 2011; Xie et al. 2025). The same applies to small‐size proteins and peptides, growth factors, enzymes with antioxidant capacity and cytokines, a diverse family suggested as biomarkers associated with semen traits as well as with (in)fertility in livestock (Alvarez‐Rodriguez et al. 2021; Barranco et al. 2021; Barranco, Rubér, Perez‐Patiño, et al. 2015; Barranco, Tvarijonaviciute, Perez‐Patinõ, et al. 2015; Parrilla, Martinez, Gil, et al. 2020; Rodriguez‐Martinez et al. 2021; Xie et al. 2025). Antioxidants present in SP, such as glutathione, ascorbate, pyruvate, taurine and vitamin E (α‐tocopherol) protect spermatozoa against deleterious levels of reactive oxygen species (ROS), as reflected in the fertility of processed ejaculates (Barranco, Tvarijonaviciute, Perez‐Patinõ, et al. 2015; Gaitskell‐Phillips et al. 2020). In addition, > 3% of SP‐proteins are antioxidant enzymes, as superoxide dismutase (SOD), gamma‐glutamyl transferase (GGT), catalase (CAT), Glutathione peroxidase (GPx), phospholipid hydroperoxide glutathione peroxidase, glutathione reductase, S‐transferase and paraoxonases (Parrilla, Martinez, Gil, et al. 2020). These enzymes, whose levels follow the highest sperm numbers in species with fractionated ejaculates (such as stallion, boar, human), help maintain ROS concentrations at physiological levels, and some are significantly associated with male fertility (Rodríguez‐Martínez et al. 2011). Such relationship is also found for the epididymal lipocalin‐type PGD2 synthase in stallions and boars, as well as paraoxonase‐1 (PON‐1) (Barranco, Tvarijonaviciute, Perez‐Patiño, et al. 2015) and lactate dehydrogenase isoenzyme LDH‐C4 in boars (Martinez et al. 2021). Unfortunately, albeit measurable we still lack standardized kits for commercial use (Bustamante‐Filho et al. 2022). The initial transitory inflammatory reactions following semen deposition (Rozeboom et al. 2000; Schjenken and Robertson 2020) and the transcriptomic response of the uterine tissue differ depending on the fertility of the sire (Donnellan et al. 2023). The response can be elicited by specific SP‐proteins (as PSP‐I/II) recruiting signals for immune cells (Rodriguez‐Martinez et al. 2021) or by short‐lived SP‐cytokines upregulating the secretion of proinflammatory cytokines by the female genital tract (Barranco, Rubér, Perez‐Patiño, et al. 2015). These early events of immune activation are thought to drive the differentiation of T‐cells into regulatory T‐cell (T_reg_) in human (X. Zhang, Greve, et al. 2024) and other species (Martinez and Rodriguez‐Martinez 2022), leading to a localized immunological shift from inflammation towards anti‐inflammatory pathways promoting the establishment of a long‐lasting period of maternal tolerance to paternal epitopes that can persist throughout pregnancy and beyond (Martinez et al. 2020, 2022; Martinez and Rodriguez‐Martinez 2022; Parrilla, Gil, et al. 2022; Parrilla, Martinez, Cambra, et al. 2020; Parrilla, Martinez, Gil, et al. 2020; Parrilla, Rodriguez‐Martinez, et al. 2022; Xie et al. 2025). These effects have been ascribed to many components of the SP, including TGF‐ß (O'Leary et al. 2011) and PGEs (Rodriguez‐Martinez et al. 2022), which promote the differentiation of T‐cell towards Th2 (IL‐4 secreting) and Th3 (IL‐10 secreting) (Robertson et al. 2002). The family of leptin and adipose tissue‐derived adipokines in SP might contribute to these immunomodulatory effects in both animals and human (Rodríguez‐Martínez et al. 2011; Rodriguez‐Martinez et al. 2021).

Many, if not most free SP‐molecules have a relative short‐life in vivo, being either eliminated, degraded (by proteases or nucleases) or metabolized. Some, as SP‐polyamines, substrates (fructose, glucose, citrate), prostaglandins or membrane lipids provide essential energy for spermatozoa (Rodriguez‐Martinez et al. 2024). Metabolomic studies have further revealed some molecules as biomarkers for either semen quality and/or fertility, including even litter‐size (Llavanera 2024; Mateo‐Otero et al. 2021).

## 3 The Particulate SP: Extracellular Vesicles

The SP is not only a complex mixture of the short‐lived free molecules described above. Large numbers of nano‐sized cell‐derived proteolipid membrane‐bound sEVs delivered by epithelial cells along the male genital organs are also conspicuously present (Skalnikova et al. 2019). These membrane‐bound sEVs encapsulate a diverse cargo of fragile biological components, i.e., lipids, signalling proteins, small non‐coding and regulatory RNAs (ncRNAs) (Golchin et al. 2026; Jeppesen et al. 2024; Roca et al. 2022), protected from enzymatic degradation.

Seminal vesicle‐like membranous structures, first reported in rabbits in 1968 were subsequently identified in human, ram, bull, stallion and boar semen between 1980 and 2000 (Roca et al. 2022), becoming a steady source of research information. Solely comparing numbers of publications listed by PubMed (2026) between 1957 and 2026, those regarding semen listed > 56,000, those related to spermatozoa > 144,000 and those for SP > 59,000. In contrast, publications on sEVs were 347 for the period 1980–2026, most (192) between 2005 and 2026. Deeper literature comparative surveys on EVs in relation to fertility were performed by (Parra et al. 2023) with an update by (Martínez‐Díaz, Parra, Montesdeoca, et al. 2024) highlighting 79 articles focusing on male reproductive EVs in pigs and bulls. Most studies relate to EVs presence and their biological roles, using mostly ultracentrifugation (UC) as harvesting method. However, only 24% of the studies followed the recommendations of the International Society for Extracellular Vesicles (Minimal Information for Studies of Extracellular Vesicles, MISEV) (Welsh et al. 2024). Of particular interest are the results of 19 articles with a good sEV characterization, showing sEVs were phenotypically and compositionally heterogeneous, yet evidencing specific sources must be determined (Martínez‐Díaz, Parra, Montesdeoca, et al. 2024). Differences in biogenesis? Composition? Different markers? Relation to the end‐target, sperm homeostasis, or even fertility? Answering these questions is essential since sEVs might exert specific actions, owing to different cargos of proteins, lipids or nucleic acids (including the regulating small non‐coding RNAs, ncRNAs) or act (as SP molecules did) in concert, directly to spermatozoa and lining epithelia or indirectly, i.e., the cargo delivered or loaded to a spermatozoon later reaching the oocyte (Vallet‐Buisan et al. 2023).

### 3.1 EVs Types and Biogenesis

Two general EV‐classes are best defined: *exosomes* (< 150 nm), originated from multivesicular bodies (MVEs) or amphisomes, and released following fusion with the plasma membrane; and *ectosomes*, directly out‐budded from the plasma membrane and displaying a large variation in size (100 nm to 1 μm) (Meldolesi 2018). As well, there might be apoptotic bodies, large vesicles released during cell death (1–5 μm), involved in the clearance of cellular debris; or non‐vesicular extracellular particles (NVEPs). Overlapping sizes complicate the isolation and characterization of specific EV populations (Jeppesen et al. 2024). Likewise, their nomenclature is complicated by the selective and progressive incorporation of bioactive molecules along their biogenesis, complicating isolation of pure EV‐subtype samples (Ragni 2025), demanding to clarify their *heterogeneity* (biogenesis, size and membrane composition) and their *molecular load* (Jeppesen et al. 2024). Thus, the term EVs ought to be considered *generic*, including different categories of vesicles (Welsh et al. 2024).

The sEVs are evolutionarily conserved among species with internal fertilization (Roca et al. 2022). Like other EVs, they are released either by exocytosis (exosomes), out‐budding (ectosomes), or through classical apocrine secretion (Foot and Kumar 2021; Parra et al. 2024) by the epithelial cells along the male reproductive tissues, including the testis (Sertoli and other sustentacular cells in the excurrent ducts), the epididymis (epididymosomes), the prostate gland (prostasomes), the seminal vesicles (vesiculosomes), and the ampulla of the ductus deferens. As a result, the SP contains a very heterogeneous mixed population of sEVs, all with a hint of functional specialization, one might presume (Muñoz et al. 2022; Parra et al. 2024; Roca et al. 2022; Martinez et al. 2021). There is, and articles related to sEVs are no exception, a rather wide liberal use of the term exosome (Martínez‐Díaz, Parra, Sanchez‐López, et al. 2024). In the case of sEVs, they are classed as small or large (size below or above 200 nm) (Alvarez‐Rodriguez, Ljunggren, et al. 2019; Barranco et al. 2019; Parra et al. 2024; Ragni 2025), due to the relative easiness to separate them and the difference in function they seem to display (Barranco et al. 2023; Dissanayake et al. 2024; Golchin et al. 2026; Parra et al. 2024; Tamessar et al. 2021, 2024).

### 3.2 Seminal EVs Isolation

Although the SP can be easily separated from spermatozoa and other cells by centrifugation or gradient separation, the isolation of the sEVs is more challenging, owing to their low buoyant density and their varying nanoscale size, requiring several methods including UC, ultrafiltration (UF), size exclusion chromatography (SEC), capture by immunoaffinity (IAC), polymer‐based precipitation and use of microfluidics (MF) (Gad et al. 2025; Jeppesen et al. 2024; Martínez‐Díaz et al. 2025; Ragni 2025). The UC methods (differential‐UC and density gradient‐UC) are most widely used to separate sEVs by size and density. However, while differential‐UC is rather raw and leads to co‐isolation of contaminants and structural damage, density‐gradient UC provides higher purity and less damage but is very cumbersome and gives low yields (Gad et al. 2025; Ragni 2025). Ultrafiltration (UF) uses membrane filters to separate EVs by size and molecular weight. It is quicker than UC, but can lead to vesicle damage and membrane clotting (Jeppesen et al. 2024). SEC‐eluting technique isolates EVs based on size, providing large yields while preserving vesicle integrity and function and benefits if associated with previous differential centrifugations (Martínez‐Díaz et al. 2025). The IAC targets specific EV‐surface proteins using antibodies or ligands attached to magnetic beads, allowing the separation of distinct EV subpopulations while maintaining EV function, but yield is low if markers are not fully expressed. This method improves if combined with UC or SEC (Jeppesen et al. 2024). Precipitation using polyethylene glycol (PEG) aggregates and precipitates EVs, becoming user‐friendly and simple, ideal for clinical settings. However, this technique is often associated with low purity due to contamination with free proteins (Gad et al. 2025; Ragni 2025). MF‐commercial devices integrating size‐based, immunoaffinity and dynamic separation are probably the best in terms of yield purity but solely from small sample volumes. They are further burdened by costs, advanced expertise and equipment. In sum, each method has pros and cons (Gad et al. 2025; Jeppesen et al. 2024; Ragni 2025), so combinations as, i.e., SEC with repeated previous centrifugations (up to 20,000xg (Martínez‐Díaz et al. 2025)) balance integrity, yield, purity and function.

### 3.3 Characterization Methods

Once isolated, the EV‐samples should be analysed to characterize the EV population. According to the MISEV guidelines, multiple, combined and complementary approaches should be used to analyse the phenotypic characteristics of EVs including concentration and particle size, EV morphology and EV protein markers. Nanoparticle tracking analysis (NTA), which measures Brownian motion using laser light scattering microscopy, is a recommended technique to measure concentration and particle size, if ranging 30–1000 nm since the software can determine the size and concentration of nanoparticles in large samples and during real‐time analysis (Ragni 2025). Resistive pulse sensing (RPS) detects changes in resistance as particles pass through a microscopic aperture and quantifies particles ranging from 50 to 1000 nm. Cryogenic electron microscopy (Cryo‐EM) is preferable to conventional TEM for examining EV morphology because it avoids the artefacts caused by fixation and dehydration during TEM (EVs showing cup‐shaped forms), as reported in pig or chicken sEVs (Barranco et al. 2019; Cordeiro et al. 2021). Scanning electron microscopy (SEM) can also be used to observe the surface of EVs, but cryo‐EM is preferable because it allows visualization of the protein corona surrounding the EVs (Barranco et al. 2025; Parra et al. 2024). For EV protein markers, MISEV recommends identifying at least one transmembrane protein, such as tetraspanins CD9, CD63 or CD81, and one cytosolic protein, such as heat shock proteins HSC70 or HSP90. To analyse NEVPs, MISEV recommends to analyse lipoproteins abundant in the native fluids, such as albumin or apolipoproteins A1/2 (Théry et al. 2018). Western blotting (WB) is the most common technique used for these analyses despite just providing a bulk measurement. Currently, techniques that allow for single‐particle analysis such as flow cytometry (FC), are gaining popularity. Flow cytometers capable of analysing particles as small as 40–50 nm should be used. Using high‐sensitivity FC in boar SP has identified different subtypes of sEVs, differing in size, RNA‐contents (Barranco, Almiñana, et al. 2024) and protein markers and showing relative abundance differences among ejaculate fractions (Barranco, Alvarez‐Barrientos, et al. 2024).

### 3.4 EVs Molecular Composition

Among the molecules detected in or associated with EVs and characterized using MISEV‐procedures are proteins, DNA, RNA, lipids and metabolites. The composition and eventual function of this cargo depend on the originating cell and on the target cell where its effects are localized. For instance, epididymosome cargo participates in sperm maturation (Rodriguez‐Martinez et al. 2022) but they also constitute a component (albeit minor) of sEVs; do they thus retain functional roles after ejaculation?

The sEV proteome is specifically encoded by its cell source. Therefore, sEVs depict structural and associated outer proteins at the plasma membrane or on the external protein corona that surrounds it, participating in receptor‐ligand recognition and interaction with other proteins (including cytokines and enzymes), lipids, and glycans (Sengupta et al. 2026). These proteins include structural ones (mainly actin), enzymes, intracellular ion channels, and spermadhesins, as well as integrins that regulate cell proliferation, survival, and migration. They also include scaffold proteins, such as ALIX and Syntenin, essential for EV biogenesis and cargo loading, and lectins (C‐type lectins, Siglecs, galectins) which recognize and bind glycans; molecules influencing cell communication and immune regulation/responses (Ragni 2025). However, most attention has been paid to the transmembrane protein family of tetraspanins (e.g., CD9, CD63, CD81) because of their abundance in EVs, their marker capacity (Jankovičová et al. 2020), and their role in facilitating the selective binding of EVs to target cell membranes (Gurung et al. 2021). Tetraspanin‐enriched microdomains (TEMs) facilitate EV docking and selective uptake of EV cargo by target cells (Jankovičová et al. 2020). Likewise, tetraspanins have been considered as complement regulatory proteins during fertilization (Jangid et al. 2023). Lastly, proteins build the two‐layered corona, with an inner hard layer of high‐affinity proteins that are irreversibly adsorbed and display a stable composition, and an outer soft layer of low‐affinity proteins with reversible and dynamic interaction, pertaining influence from the environment (Heidarzadeh et al. 2023; Parra et al. 2024). These corona proteins seem to represent a camouflage mechanism for EVs, influencing their cellular uptake and, consequently, their half‐life (Liam‐Or et al. 2024) as well as the storage of the hormone oxytocin in pig sEV‐protein corona at concentrations correlated with the fertility of boars used in commercial AI programmes (Parra et al. 2025).

There is also a setup of inner proteins in the EV‐cargo load which reflects the cells of origin. Among these we often find heat shock proteins, Alix or TSG101, commonly identified in exosomes. The number of proteins can range from a few hundred in exosomes to more than double that amount in larger sEVs from ram semen (Leahy et al. 2020). Similar differences in protein content between large or small sEVs have also been reported in boar, where a total of 1034 proteins have been identified (Barranco et al. 2023). The proteome of epididymal EVs in livestock is differentially abundant between segments, with some proteins related to the sequential maturation of spermatozoa, specifically the acquisition of motility and capacity to bind to the zona pellucida (ZP) and oocyte fertilization (Roca et al. 2022). These processes may ultimately influence IVF results through the modulation of sperm metabolism (Barranco, Spinaci, et al. 2024). The sEVs contain immunoregulatory proteins, such as TGF‐β isoforms 1–3 (Barranco et al. 2019). Other proteins are RNA‐binding (RBPs) mediating the loading of miRNAs and other RNAs (mRNAs, lncRNAs, tRNAs, piRNAs). The amount of these RNAs vary largely among livestock sEVs and has been associated with spermatogenesis, fertility, litter size, immune function and epigenetic inheritance; linking systemic metabolic conditions to transgenerational reproductive outcomes (Roca et al. 2022; Sengupta et al. 2026; Wang et al. 2025). Small and large pig sEVs show up to 735 RNAs with large sEVs carrying most ‘all’‐RNAs, whereas miRNAs were generally more abundant in small sEVs (Barranco, Almiñana, et al. 2024). The family of miRNAs is probably the most studied load component of EV cargo, including identification of single‐ or groups of miRNAs involved in sperm cryopreservation in pigs (Pedrosa et al. 2021). In addition, piRNAs contained in sEVs are increasingly recognized as regulators of genome stability in spermatozoa and early embryos (Perillo et al. 2023). DNA is also present in EVs, either as ssDNA, mtDNA or repetitive transposons (Ragni 2025; Sengupta et al. 2026). Lipids (glycerophospholipids, sphingolipids and cholesterol) are obviously also present in EVs, being the main component of the membrane interacting with target cells, where they organize as mobile rafts to facilitate signalling (Ragni 2025). The glycerophospholipid phosphatidylserine and the abundant sphingolipid ceramide are essential in cell communication, while cholesterol appears relevant during membrane fusion (Xie et al. 2025). Small and large boar sEVs differ in lipid profile, indicating differences in functional roles. For instance, large sEVs (containing a higher proportion of ectosomes than exosomes and a higher amount of ceramide) would be able to modulate sperm metabolism (Martínez‐Díaz, Parra, Sanchez‐López, et al. 2024) and even IVF‐outcomes (Barranco, Spinaci, et al. 2024). Alongside lipids and proteins, EVs also contain glycans, such as proteoglycan (PG), heparan sulphate PG (HSPG), glycolipids and glycoproteins, all essential for EV binding to target cells and for cell communication (Ragni 2025; Sengupta et al. 2026).

In summary, the heterogeneous mixture of sEVs, produced by different cells, differs in contents and probably targets different cells. An example of such differentiation was recently shown in the boar ejaculate, with differences in the distribution of tetraspanin markers between small and large sEVs as well as among specific ejaculate fractions. In particular, vesicular‐rich samples (post‐SRF) exhibit higher proportions of CD9‐ and CD63‐positive sEVs (Barranco, Alvarez‐Barrientos, et al. 2024). Noteworthy, the hyaluronan receptor CD44 was present in all sEVs subpopulations, confirming previous studies (Alvarez‐Rodriguez, Ljunggren, et al. 2019) and being a possible universal protein marker for the entire sEVs pig population (Barranco, Alvarez‐Barrientos, et al. 2024). This widespread distribution may be related to the persistent presence of epididymosomes along the ejaculate, despite being proportionally higher in the first 10 mL of the SRF (Alvarez‐Rodriguez, Ljunggren, et al. 2019). In any case, considering hyaluronan involvement in the colonization of the oviductal sperm reservoir and the targeting of the oocyte vestments (Rodriguez‐Martinez et al. 2016), the implications of such wide distribution of CD44 in boar sEVs, being a cell surface protein important in cell‐to‐cell interaction and adhesion, appear worth further, and ongoing, investigation (P. Panales, personal communication).

## 4 Male EVs as Molecular Modulators of Function

Male EVs, either those delivered alongside the post‐testicular excurrent ducts, mainly ducti epididymides, and those finally present in the SP are able to interact (i.e., add, fuse, and internalize its molecular cargo) with specific cell targets. Through these mechanisms, EVs act as information carriers to spermatozoa (Roca et al. 2022) transferring molecules such as small ncRNAs, antioxidant enzymes and hormone receptors (e.g., for androgens, oestrogen or insulin) relevant for sperm maturation and following events related to progressive motility and capacitation (Dlamini et al. 2023; Fazeli et al. 2025; Governini et al. 2022, 2025; Panales et al. 2026). Male EVs interact with epithelial cells of the ducti epididymides (Rodriguez‐Martinez et al. 2022; Xu, Zhang, et al. 2024) but also, and perhaps more importantly, with cells of the female genital tract, where they deliver specific ncRNAs and cytokines that may influence reproductive processes (Aleksejeva et al. 2022). But, how does the uptake of molecules from the EVs occur after binding to the surface of the target cell? Several pathways are considered as membrane fusion, clathrin‐dependent endocytosis, caveolin‐mediated endocytosis, pinocytosis, phagocytosis, and lipid raft‐mediated internalization (Ragni 2025; Sengupta et al. 2026). Direct membrane fusion seems the simplest mechanism for the transfer of both membrane‐ and internal cargo to spermatozoa (Caballero et al. 2013) or lining epithelial cells (Ragni 2025). This process involves proteins such as annexins, which play a key role in membrane fusion (Schmaltz et al. 2025). Other more complicated mechanisms, as pinocytosis and phagocytosis, relate to immune cells (APCs and macrophages) that are highly efficient to engulf EVs, particularly when they express phosphatidylserine (PS) on their membrane, a well‐known marker for sperm exocytosis (Peña et al. 2003) and sperm interactions with sperm reservoirs (Schmaltz et al. 2025).

### 4.1 Epididymosomes and Sperm Maturation

The interactions of epididymosomes with the maturing spermatozoa have been extensively reviewed in animal models, for their role to provide spermatozoa with molecules promoting acquisition of sperm forward motility, oxidation–reduction, metabolism, capacitation, acrosome reaction and capacity to fertilize (Ali et al. 2023; Rodriguez‐Martinez et al. 2022; Xie et al. 2025). The role of epididymosomes might not end when spermatozoa are finally stored in the epididymal cauda, as its fluid follows sperm and epididymosomes during emission. In species with fractionated ejaculates, the first portion, often called the sperm‐peak portion, contains the first sperm load from the cauda epididymidis which, subsequently mixed with prostatic fluid, is also first to enter the female reproductive tract and be overrepresented in the oviductal sperm reservoir (Rodriguez‐Martinez et al. 2024). Since sEVs are tethered to the sperm membrane and either simply attached or being fused/internalized, it is possible that epididymosomes can modify those ejaculated spermatozoa most likely involved in fertilization, either in vivo (Barranco et al. 2026; Rodriguez‐Martinez et al. 2024) or in vitro (Luo et al. 2024).

### 4.2 Seminal EVs Effects on Ejaculated Spermatozoa

Emitted sEVs of epididymal origin alongside caudal spermatozoa are, during ejaculation, further extended with sequential secretions from the accessory sexual glands and their particular sEVs (prostasomes, vesiculosomes, etc.) some of which shall eventually bind, fuse and deliver their content to the spermatozoa (Roca et al. 2022; Rodriguez‐Martinez et al. 2024). However, not all sEVs fuse with spermatozoa, some attach and detach and some continue free in SP (Leahy et al. 2020) to influence the female epithelial lining (Barranco et al. 2020). Selective sEV‐sperm binding would also occur at different female genital compartments, depending on where the semen is deposited and the type of ejaculate of the species considered (Aalberts et al. 2013; Han et al. 2023, 2024; Roca et al. 2022; Rodriguez‐Martinez et al. 2022, 2024). The effects of sEVs on sperm attributes can vary. sEVs display a protective role against oxidative sperm DNA damage (Ribas‐Maynou et al. 2025). Small‐ and large‐sized boar sEVs can interact with fresh boar spermatozoa decreasing oxidative stress and mitochondrial activity (Parra et al. 2025). Looking at cryopreserved epididymal boar spermatozoa, sEVs seem to affect motility, but neither capacitation nor acrosome reactivity or IVF parameters (Moya‐Fernández et al. 2025). In contrast, sEVs have been shown to stabilize sperm membranes in ejaculated pig spermatozoa, thereby preventing premature capacitation and subsequent acrosome exocytosis (Du et al. 2016; Piehl et al. 2013) as well as in epididymal spermatozoa incubated with small or large sEVs, where both subtypes act in a complementary way to regulate sperm capacitation (Panales et al. 2026). Ejaculated sperm incubation with sEVs improved total and progressive sperm motility in dogs, possibly via transfer of Zn^2+^ ions (Mogielnicka‐Brzozowska et al. 2015) but not in human, with only subtle increases in progressive motility at pH 7.0 (Tamessar et al. 2024) nor stallions, where capacitation was not affected (Aalberts et al. 2013), but where differences in cryotolerance were related to the amount of smaller and more functional sEVs they were exposed to (Barranco et al. 2025). Sperm motility seems influenced by the uploading of molecular sEV components activating CatSper‐mediated calcium signalling (Zhang, Liang, et al. 2024; Zhang et al. 2021). In addition, sEVs may enhance motility through the transfer of extra ATP produced in sEVs (Guo et al. 2019). Differences in these effects are evident among ejaculates with different quality (Dlamini et al. 2023), particularly motility (Zhao et al. 2024). In cryopreserved spermatozoa, variability can reside in differences in freezing/thawing methods (Roca et al. 2006) as well as with sperm source, especially in species with fractionated ejaculates (Parra et al. 2024). In any case, it seems evident that sEVs protect sperm mitochondrial and plasma membranes (Kowalczyk and Kordan 2024). However, these effects are less pronounced than those exerted by EVs of female origin (uterosomes/oviductosomes), as reported in both pig (Alcântara‐Neto et al. 2020; Fang et al. 2023; Roca et al. 2020, 2022, 2006) and human (Fasoli et al. 2025).

### 4.3 Seminal EVs Effects on the Female Internal Reproductive Tract

Following semen deposition, it is assumed that sEVs would bind to and internalize their cargo in epithelial cells lining the internal tract (Paktinat et al. 2019). They can also alter gene expression in bovine oviductal epithelial cells (BOEC) in vitro, leading to alterations in the small ncRNAs contained in their oviductomes, e.g., their own produced EVs (Reshi et al. 2023). These ncRNA cargos may play essential roles in pre‐ and post‐fertilization events, as miRNAs present in the uterine lumen appear to modify bovine early embryo development (Welsh et al. 2024) as well as fertility (Ishikawa et al. 2024). Even circular RNAs (circRNAs) expressed in ovine sEVs appear to regulate specific miRNA pathways in endometrial epithelial cells in vitro related to embryo implantation (Xie et al. 2026). Years ago, it was reported that boar sEVs had the capacity to increase the production of proteins related to immune and inflammatory responses in pig endometrial epithelial cells in vitro (Bai et al. 2018). Likewise, there is evidence that the SP, and possibly sEVs, promote the recruitment of PMNs during the transient uterine inflammatory reaction following semen deposition (Martinez et al. 2021). In addition, the testicular glycosylated glycophosphoinositol‐anchored peptide CD52 present in human sEVs has been shown to mediate T cell suppression indirectly via Siglec‐7 on APCs (Harrison et al. 2024). Additional inseminations with homologous/pools of SP improves rates of early embryo survival, implantation, placental development, farrowing rates, litter size and even offspring development and health in livestock, pigs in particular (Martinez et al. 2021), effects conveyed, most likely, by changes in immune responsiveness by the female (Schjenken and Robertson 2020). Boar SP can induce changes in gene expression by the internal female genitalia (Alvarez‐Rodriguez, Atikuzzaman, et al. 2019; Alvarez‐Rodriguez et al. 2024; Martinez et al. 2019, 2020), and over activate the TGF‐ß T‐signalling pathway, increasing the development of T_reg_ cells and helping control the immune response of the female to the presence of hemi‐allogeneic embryos (Martinez and Rodriguez‐Martinez 2022). The seminal TGF‐ß isoforms involved in these effects may, at least in part, be released via sEVs in pigs (Padilla et al. 2023). Both SP and TGF‐ß1 were able to influence endometrial cytokines; however, only SP impacted pre‐implantation pig embryo development (Martinez et al. 2021). This finding suggests that SP exerts via various factors a concerted modulating action towards embryo development by modifying gene expression in the female endometrium. These changes include the regulation of large number of small ncRNAs, including miRNAs (9%, almost 300 variants) having important roles in immune regulation (Barranco et al. 2020; Roca et al. 2022; Rodriguez‐Martinez et al. 2022), sperm motility (Martin‐San Juan et al. 2026) and fertility (Alvarez‐Rodriguez et al. 2020). Among these, specific miRNAs such as ssc‐miR‐10b have been implicated in reproductive processes (Xu et al. 2020; Xu, Xie, et al. 2024).

Do sEVs also interact with the oocyte and its vestments? It is evident that sEVs modulate gene expression in pig cumulus cells under in vitro conditions (Mateo‐Otero et al. 2022). In vivo, specific SP‐proteins as the spermadhesin AWN‐1 have been detected in the outer ZP of pig zygotes (Rodríguez‐Martínez et al. 1998) so sEVs can be possibly involved. Likewise, CD44 is present in pig sEVs, irrespective of the fractions studied (Barranco, Alvarez‐Barrientos, et al. 2024). It can thus be argued that sEVs can bound to the hyaluronan present among cumulus cells, alternatively that sperm‐sEVs fusion incorporated such CD44. Either way it can be speculated that the CD44‐HYA recognition is a remarkable step in reaching the ovulated COCs in the vast environment of the oviductal ampulla segment, and experimental testing is foreseen, or at least, wanted. Do sEVs penetrate the pores of the ZP and do some components as scRNAs reach directly to the oocyte? Not known nor tested yet, to the best of our knowledge. However, the question is valid considering the differences seen between in vivo and in vitro, where some of the physiological hurdles for fertilization are modified (i.e., cumulus cells removal, sperm cleansing). Moreover, signalling for altered phenotype (e.g., epigenetic driven) might also originate from SP‐components (Roca et al. 2022) as supported by rodent experiments where miRNas from epididymosomes of stressed males injected into unstressed zygotes yield offspring with stressed phenotypes (Wang et al. 2021).

## 5 Seminal EVs and Fertility: Biomarkers or Effectors?

The SP has largely been made responsible to facilitate fertility in vivo (Martinez et al. 2021) with SP‐profiling suggested to estimate the fertility potential of a given male (Pérez‐Patiño et al. 2018). However, such an approach is coarse and further blurs the potential roles of specific components. Markers for sperm dysfunction and (in)fertility were detected in spermatozoa and SP of many species, including human and livestock, including fertility‐related proteins (Pal et al. 2025), anti‐oxidative enzymes, metabolites, and miRNAs (Martin‐San Juan et al. 2026; Roca et al. 2020; Rodriguez‐Martinez et al. 2024; Xie et al. 2025). Yet can sEVs also provide those biomarkers?

### 5.1 Seminal EVs Biomarkers for Sperm Function and Fertility

Studies during the past few years (Xie et al. 2025) have shown that sEVs provide biomarkers for fertility in livestock (bull, boar) mostly in the shape of proteins and/or miRNAs. Sperm motility is related to various sEV cargoes, such as miRNAs, mRNAs, proteins, metabolites (Yu et al. 2023) and lipids. Lipidomic analyses of sEVs from boars with different sperm motility identified important lipids and genes (*ATP2B1*) regulating metabolic pathways, such as glycerophospholipid metabolism and ferroptosis pathways, thus influencing motility (Ding et al. 2025). A further example is the finding that sEVs from roosters with high‐ respectively low‐ sperm motility have different miRNA expression profiles that mark a number of 34 miRNAs load as suitable biomarkers for sperm motility with sEVs addition to fresh and frozen ejaculates improving fertility (Chen, Xie, et al. 2025; Xu, Zhang, et al. 2024), indicating gga‐miR‐24‐3p as the responsible (Han et al. 2024). Studies on other species, as porcine, have shown that the load of miRNAs in sEVs derived from ejaculates with divergent quality reveals significant differences, in relation to sperm motility regulation (Dlamini et al. 2023, 2025; Zhao et al. 2024) identifying groups of miRNAs in sEVs from AI‐doses (miR‐6529‐5p, miR‐146a‐5p) as potential markers for high sperm motility (Martin‐San Juan et al. 2026). Likewise, miRNAs in small sEVs (ssc‐miR‐122–5p, ssc‐miR‐486, ssc‐miR‐345‐3p, ssc‐miR‐362, ssc‐miR‐500‐5p, miR‐15a‐5p) have been connected to low motility (Martin‐San Juan et al. 2026; Zhao et al. 2024) or low cryotolerance (ssc‐miR130a, ssc‐miR‐9) (Pedrosa et al. 2021). miRNA‐detection in small sEVs has also been related to fertility, both in bovine (bta‐miR‐195, Chauhan et al. 2024) and porcine (miR‐26a, Chen, Zheng, et al. 2024) among other species (Xie et al. 2025). Specific proteins, such as Ezrin, negatively affects fertility, at least in vitro (Xu, Zhang, et al. 2024). As well, proteomic studies of sEVs derived from buffalo bulls with different sperm motility have shown differential expression of aquaporin AQP7, a protein essential for sperm membrane function, which is significantly lower in low‐motility bulls (Yu et al. 2023), potentially compromising even sperm preservation. Proteins from sEVs (both from small, but mostly from large sEVs) may well be involved in boar fertility, mainly through modulation of sperm function (as Notch3, Muc6, Pigr, St3gal1, Rnase9), ZP‐binding (as Adam5, Bpi, Spaca4, Atp8b3, Glipr1l1, Afp) but also related to embryo development and implantation (Ace, St6galnac1), as well as in relation to the immune system (Hpn, Entpd6, Adam9) (Barranco et al. 2026). In this very recent study, the proteome of sEVs derived from 18 boars with the greatest differences in fertility revealed that of these 16 sEV‐proteins, five of them were differentially expressed between boars with high and low farrowing rates and 15 proteins differed between sires with larger vs. smaller litter sizes, variables that account for fertility in polytocous species. The most fertile sires held some proteins while the least fertile boars held another set, exclusively (Barranco et al. 2026) suggesting a mapping of these proteins, which are transmembrane or membrane proteins in the outer surface of the EV membrane or extracellular proteins associated to the sEVs corona, acting as either signalling proteins (Cerrotti et al. 2024) or cell adhesion proteins, can provide convenient biomarkers. The rationale behind is that these proteins are those detectable using FC‐immunophenotyping without disrupting the sEVs. This comparative proteome analysis can further be applied at commercial settings, since they can be mapped (Barranco, Alvarez‐Barrientos, et al. 2024) and be included in fertility predictive models aiding the selection of most fertile sires for AI‐use. The selective power and the economic gains of selecting or refusing males delivering semen for AI before their use in the field by predicting their fertility beforehand at production level, are very appealing. In sum, sEVs cargo can apparently biomark potential sire fertility but, can cargo be modified to alleviate semen handling or improve ARTs?

### 5.2 Using sEVs to Improve Fertility

Some time ago, we reviewed available information regarding the potential use of sEVs from highly fertile males during IVP or even during AI (Martinez et al. 2021) mimicking results obtained using SP from good‐freezers as additive during freezing/thawing (Roca et al. 2006). Further studies have been directed to specific either free‐ or particulate (sEVs) components (Xue et al. 2024), proteins with antioxidant properties (Ribas‐Maynou et al. 2025), and specific cytokines (Garcia‐Canovas et al. 2026), with encouraging outputs. What might be the next step? Increasing the load of specific components in natural sEVs? Or simply building tailored sEVs using liposomes? Obviously, there are pros and cons. For example, about 93% of female pigs are inseminated with liquid semen at 40 mL doses (Mellagi et al. 2023), which constrains the practical use of added sEVs to such volumes. Alternative semen deposition strategies, such as deep intrauterine‐AI (Martinez et al. 2001), may help overcome some of these limitations.

## 6 Pros and Cons of Using sEVs for AI, IVF, and Other ARTs

Examples for the enrichment, modification and even creation of specific sEVs for use, preliminarily in vitro, have been thoroughly reviewed (Chen, Zheng, et al. 2024; Rodriguez‐Martinez and Roca 2022). The processes imply either the collection, characterization and conservation of natural sEVs from highly fertile males for direct use as additives, or the use of sEVs from any male that can be subsequently enriched or modified using various methods (Dave et al. 2025; Sadeghi et al. 2023). Such approaches aim to ‘improve’ their performance as sperm function or fertility promoters (Franko and de Almeida Monteiro Melo Ferraz 2024). These strategies can be readily implemented in species with abundant sEVs, as they can be easily characterized, handled, preserved and modified. Examples in livestock are logical, and they can be extended to humans, respecting ethical regulations (Wang, Tsai, and Lee 2024). Last but not least is the production of ‘artificial’ sEVs, using liposomes ‘filled’ with a desired cargo perhaps present in small or large sEVs (Hadizadeh et al. 2022; Rodriguez‐Martinez and Roca 2022; Sadeghi et al. 2023). Even if this production is feasible, our level of knowledge of the synergies related to cargo loads, and the relation between sEVs and the target cells, whether spermatozoa, epithelial lining of the female genital tract or specific receptor cells (as APCs) is yet to be fully disclosed, since we cannot rule out negative effects on the immune system (Wang, Li, et al. 2024).

Worth is to note that all the above handling and eventual manipulation is to be done in vitro, where all this biological material is exposed—since a long time—to plastic ware. Nanoplastics have been detected in body fluids (including semen, and hence its components), follicular fluid, and oocytes (Kong et al. 2025) but even EVs (Calzoni et al. 2024). Far are we from grasping the consequences of nanoplastics on gamete function and embryo development, although some risks for undesirable components have been listed (Winiarska et al. 2024). Therefore, any further handling in vitro would maintain and even possibly increase the risk by plastic contamination already present in sires during procedures, demanding caution.

## 7 Final Remarks

Overwhelming evidence, both clinical and experimental, indicates that the SP and its particulate component of sEVs play critical roles in preserving sperm homeostasis, even in handled or cryopreserved, facilitating sperm‐oocyte binding and pregnancy of the alloantigenic embryos by modulating the maternal immune system to a state of tolerance. Major efforts have been made to isolate which molecules could be of value to improve fertility under commercial settings, whether proteins or ncRNAs, free or contained in sEVs of either high fertility sires or even in laboratory‐produced EVs. Differences in the sEVs derived from males with significantly different fertility underpin their potential as non‐invasive diagnostic tools (Dlamini et al. 2025; Rana et al. 2024), including the mapping of identifiable sEV fertility‐relevant proteins among sires with highest/lowest fertility (Barranco et al. 2026) which can beforehand aid prediction of fertility post‐AI at production level. Many are the challenges ahead to provide commercial breeders with economically viable novel instruments to increase fertility on‐farm. New generations of researchers are on this track, godspeed!

## Author Contributions

H.R.M. conceptualized the manuscript and wrote the original draft. All co‐authors (J.R., F.P.V., E.A.M.) reviewed and edited the manuscript. All authors acquired external funding for the reported own studies.

## Funding

The research of the authors was funded by The Spanish Ministry of Science, Innovation, and Universities, and the European Regional Development Funds: MICIU/AEI/10.13039/501100011033 and ERDF/EU (PID2022‐137738NA‐I00; PID2022‐137645OB‐I00; PID2024‐159343OB‐I00). Fundación Séneca (Murcia, Spain): FSRM/10.13039/100007801 (21935/PI/22 and 23014/GERM/25).

## Ethics Statement

Animal husbandry and experimental procedures, when applicable to the studies reported here by the authors, were done in accordance with the current European Directive 2010/63/EU, 22/09/20, and Swedish (SJVFS 2017:40) and Spanish (ES300130640127) legislation and approved in advance.

## Conflicts of Interest

The authors declare no conflicts of interest.

## Data Availability

Data sharing not applicable to this article as no datasets were generated or analysed during the current study.
